# Hearing Loss as the Main Clinical Presentation in *NLRP3*-Associated Autoinflammatory Disease

**DOI:** 10.3389/fimmu.2022.904632

**Published:** 2022-05-26

**Authors:** Dominika Oziębło, Marcin L. Leja, Aldona Jeznach, Magdalena Orzechowska, Tomasz Skirecki, Ewa Więsik-Szewczyk, Mariusz Furmanek, Natalia Bałdyga, Henryk Skarżyński, Monika Ołdak

**Affiliations:** ^1^ Department of Genetics, Institute of Physiology and Pathology of Hearing, Warsaw, Poland; ^2^ Postgraduate School of Molecular Medicine, Medical University of Warsaw, Warsaw, Poland; ^3^ Laboratory of Flow Cytometry, Centre of Postgraduate Medical Education, Warsaw, Poland; ^4^ Department of Internal Medicine, Pneumonology, Allergology and Clinical Immunology, Central Clinical Hospital of the Ministry of National Defense, Military Institute of Medicine, Warsaw, Poland; ^5^ Bioimaging Research Center, Institute of Physiology and Pathology of Hearing, Warsaw, Poland; ^6^ Oto-Rhino-Laryngology Surgery Clinic, Institute of Physiology and Pathology of Hearing, Warsaw, Poland

**Keywords:** NLRP3 inflammasome, hearing loss, anakinra, autoinflammation, fluid attenuated inversion recovery (FLAIR), cochlear enhancement, DNA sequencing, interleukin-1

## Abstract

The *NLRP3* gene mutations are the cause of autosomal dominant autoinflammatory disorders (NLRP3-AID). Recently, hearing loss (HL) has been found to be the sole or major manifestation of NLRP3-AID. Here, we tested 110 autosomal dominant HL families with a custom panel of 237 HL genes and found one family carrying the *NLRP3* c.1872C>G, p.Ser624Arg mutation. Functional studies revealed that this novel variant is a gain of function mutation, leading to increased activity of caspase-1 and subsequent oversecretion of proinflammatory interleukin-1β. Clinical reanalysis of the affected individuals, together with serological evidence of inflammation and pathological cochlear enhancement on FLAIR-MRI images, guided our diagnosis to atypical NLRP3-AID. The study highlights the role of genetic analysis in patients with progressive postlingual HL. This can help to identify individuals with hereditary HL as a consequence of NLRP3-AID and allow timely and effective treatment with interleukin-1-receptor antagonist.

## Introduction

Pathogenic variants in single genes constitute a substantial portion of the causes of hearing loss (HL). With the progress of DNA analysis techniques and the increasing availability of genetic testing for routine clinical diagnosis, detection of these variants continues to grow. Following the diagnosis of hereditary HL, evaluation and treatment options are now available, although most are symptomatic and aimed at alleviating disease symptoms and preventing complications. But if one could identify the underlying mutation and its related molecular background, this raises the prospect of a new therapy targeting the causal mechanism itself (1).

In 2017, Nakanishi et al. reported patients from a family with syndromic progressive HL who responded to treatment with anakinra, an antagonist of the interleukin-1 (IL-1) receptor. The therapy was introduced to counteract increased secretion of IL-1β, which was a consequence of an activating *NLRP3* mutation in these patients (2). The protein encoded by *NLRP3* is a member of the NLR (nucleotide binding domain, leucine rich repeats-containing) family and the NLRP (pyrin domain containing) subfamily. It is abundantly expressed in neutrophils and macrophages and is considered a critical mediator of inflammation. Following stimulation by diverse triggers – such as endogenous stress signals, exogenous particulates, pathogens, and pore-forming toxins – NLRP3 inflammasome is assembled. The NLRP3 inflammasome promotes activation of procaspase-1 that in turn cleaves and activates the proinflammatory cytokines interleukin IL-1β and IL-18 (3).

*NLRP3* was discovered through its association with autosomal dominant autoinflammatory diseases (NLRP3-AID), also known as CAPS (cryopyrin-associated periodic syndromes), which comprise a spectrum of inflammatory symptoms (e.g. urticaria, conjunctivitis, myalgia, arthralgia, fever, headache, fatigue) of various severity (4). Progressive sensorineural HL with onset in childhood or early adulthood has been typically reported in moderate and severe forms of NLRP3-AID. HL as the sole or major NLRP3-AID associated feature is a recent and surprising finding that deserves attention, particularly since a targeted anti-IL-1 treatment is available that has been shown to be effective throughout the NLRP3-AID spectrum (5).

In this study, we present a family initially diagnosed with an isolated autosomal dominant sensorineural HL (ADHL). Based on the results of our genetic testing and targeted detailed assessment of the clinical picture, the diagnosis has now been shifted to atypical NLRP3-AID. Magnetic resonance imaging (MRI) of the temporal bones has revealed that cochlear inflammation accounts for progressive hearing deterioration, and we find that a later acquisition of the MRI-FLAIR images after contrast administration is even more sensitive in detecting the pathological cochlear enhancement. A novel *NLRP3* mutation has been identified as the only variant capable of causing the phenotype in this family. After applying a set of functional assays, we have confirmed that the NLRP3 inflammasome is hyperactive in these patients, leading to increased activity of caspase-1 and overproduction of IL-1β.

## Materials and Methods

### Study Subjects

**Figure 1:**
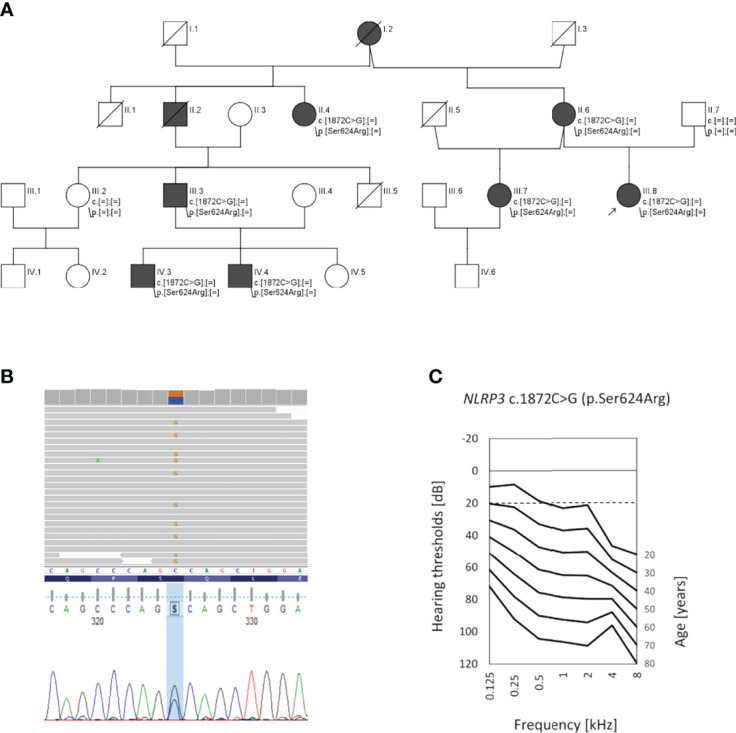
Pedigree of the investigated family, genetic data, and audiological characteristics. **(A)** Pedigree showing autosomal dominant hearing loss (black symbols); healthy individuals are shown in white. The proband (III.8) is indicated by an arrow. **(B)** Results of next-generation sequencing and Sanger sequencing showing the c.1872C>G transversion (p.Ser624Arg) in the *NLRP3* gene. **(C)** Age-related typical audiogram (ARTA) based on audiological data of the 5 patients with *NLRP3* p.Ser624Arg as the likely pathogenic variant. The normal hearing threshold is marked with a detached line.

All participants gave written informed consent. The study was approved by the local ethics committee (KB.IFPS.25/2017) and performed according to the Declaration of Helsinki. A non-consanguineous Polish family with 9 family members affected by bilateral sensorineural HL was identified at the Institute of Physiology and Pathology of Hearing. The family was selected based on genetic testing results from a cohort of 110 HL families presenting a pedigree consistent with the autosomal dominant mode of HL inheritance. In the proband (III.8), HL was noted at the age of 20; since then it had gradually progressed. The proband has used hearing aids from the age of 34. At age 10, she was treated with gentamicin for peritonitis. In her mother (II.6), hearing deterioration was diagnosed at age 37, and from the age of 38, she wore hearing aids. Additionally, she has been diagnosed with schizophrenia and has received appropriate treatment from the age of 35. The proband’s stepsister (III.7) noticed progressive HL at the age of 40, and this was confirmed by objective hearing tests at age 43. All of them suffer from tinnitus but do not report vertigo or dizziness. In individuals II.2 and II.4, hearing deterioration was first observed at the end of the fourth decade of life; in subject III.3 it was seen at around age 20. Individuals IV.3 and IV.4 have suffered from recurrent episodes of choroiditis from the age of 8, and their hearing deterioration was noticed at the age of around 15 (**Figure 1A**).

### Clinical Evaluation

Affected family members (II.6, III.7, III.8) underwent clinical evaluation by an otorhinolaryngologist, clinical geneticist, and clinical immunologist. Hearing status was assessed by standardized analysis including tympanometry, pure-tone audiometry (PTA), and auditory brainstem responses (ABR). A set of 18 binaural hearing thresholds was obtained from 5 available family members with HL (nine from patient III.8, three from patient II.6, three from patient III.7, two from patient II.4 and one from patient III.3). The age-related typical audiogram (ARTA) was constructed as described previously (6). Based on cross-sectional linear regression, hearing thresholds were predicted for fixed ages (20–80 years) and an annual threshold deterioration (ATD; dB per year) was calculated for all tested frequencies. The progression was considered significant if the regression coefficient (slope) was significantly different from 0 at p < 0.05 (GraphPad Prism 9.0).

MRI of the inner ear was performed along with brain imaging on a 3 T MRI system (Magnetom Prisma, Siemens). A protocol designed for inner ear and inner auditory canal imaging was employed; it contained heavy T2-weighted images of 0.5 mm slices (constructive interference in a steady-state (CISS) sequence) and FLAIR (fluid-attenuated-inversion-recovery with inversion time of 1674.8 ms) images of 2 mm slices acquired before and after (approx. 15 min and 75 min) i.v. administration of gadolinium contrast agent (0.1 mmol/kg of gadoteridol, ProHance; Bracco Imaging). Images were reviewed by a radiologist with over 15 years of expertise in neuroradiology.

### Pathogenic Variant Detection

Genomic DNA was extracted from blood samples or buccal swabs. For the proband (III.8), a custom multi-gene HL panel (*n*=237 genes) was used, and the subsequent data analysis workflow was performed as previously described (7). To confirm the presence of the detected *NLRP3* variant and its segregation with HL in the family, Sanger sequencing with the following primer pair encompassing exon 3 (5′-AAGGAAGTGGACTGCGAGAAG and 5′-CCACCCGATGACAGTTCTCAA) was applied as described elsewhere (8). The variant was assigned based on the reference sequences NM_001243133.1 and NP_001230062.1 according to standards and guidelines for the interpretation of sequence variants (9, 10).

### Isolation and Stimulation of PBMC

Blood samples from patients II.6, III.7, III.8 and age- andsex-matched healthy donors were collected by EDTA anticoagulant on the same day as the isolation of peripheral blood mononuclear cells (PBMCs) and assays were performed. PBMCs were isolated using Ficoll-Histopaque solution (Sigma-Aldrich, Saint Louis, MO, USA). Blood samples were diluted 1:1 with PBS (Sigma-Aldrich) and layered on top of the Ficoll solution. Cells were centrifuged for 25 min at 2000 rpm. Then the cell-containing phase was collected and washed 3 times using PBS with 2% heat-inactivated bovine serum (Sigma-Aldrich). For experiments, cells were resuspended in RPMI-1640 medium (Gibco, Thermo Fisher Scientific, Waltham, MA, USA) with 3% heat-inactivated human serum and antibiotics (penicillin and streptomycin (Thermo Fisher Scientific)). PBMCs were seeded on 96-well plates at a density of 10^5^ cells per well. Colchicine, known for its ability to suppress NLRP3 inflammasome activation (11), (10 µM; Sigma-Aldrich) or MCC950, a selective NLRP3 inhibitor (5 µM; Inflazome Ltd, Ireland), were added; 30 min later ultrapure *E. coli* lipopolysaccharides (LPS, InvivoGen, San Diego, CA, USA) were also added for stimulation. After 1 h, nigericin, an NLRP3 activator (12), was also added (10 µM; Tocris, UK) and the cells were incubated at 37°C in a humidified atmosphere of 95% O_2_ and 5% CO_2_. 3 h or 24 h after LPS addition, the supernatants were collected for the IL-1β ELISA assay or the Caspase-Glo 1 Inflammasome Assay (Promega, Madison, WI, USA) was performed.

### ELISA for Interleukin-1β

Levels of secreted IL-1β were measured in the supernatants using a human IL-1β ELISA kit (Thermo Fisher Scientific) according to the manufacturer’s protocol. The plate was read using the Multiskan GO microplate spectrophotometer (Thermo Fisher Scientific).

### Caspase-Glo 1 Inflammasome Assay

The activity of caspase-1 was measured together in whole cells and in supernatants using the Caspase-Glo 1 Inflammasome Assay (Promega) following the manufacturer’s instructions. Cells were treated on a Z-WEHD-aminoluciferin substrate for 1 h at room temperature. Luminescence was measured using a SpectraMax i3x multi-mode microplate reader (Molecular Devices LLC, San Jose, CA, USA).

### Statistics

The measures of the functional activity of the NLRP3 inflammasome were compared using *t*-tests (GraphPad Prism 9.0).

## Results

### Identification of a Novel Likely Pathogenic *NLRP3* Variant

**Figure 2:**
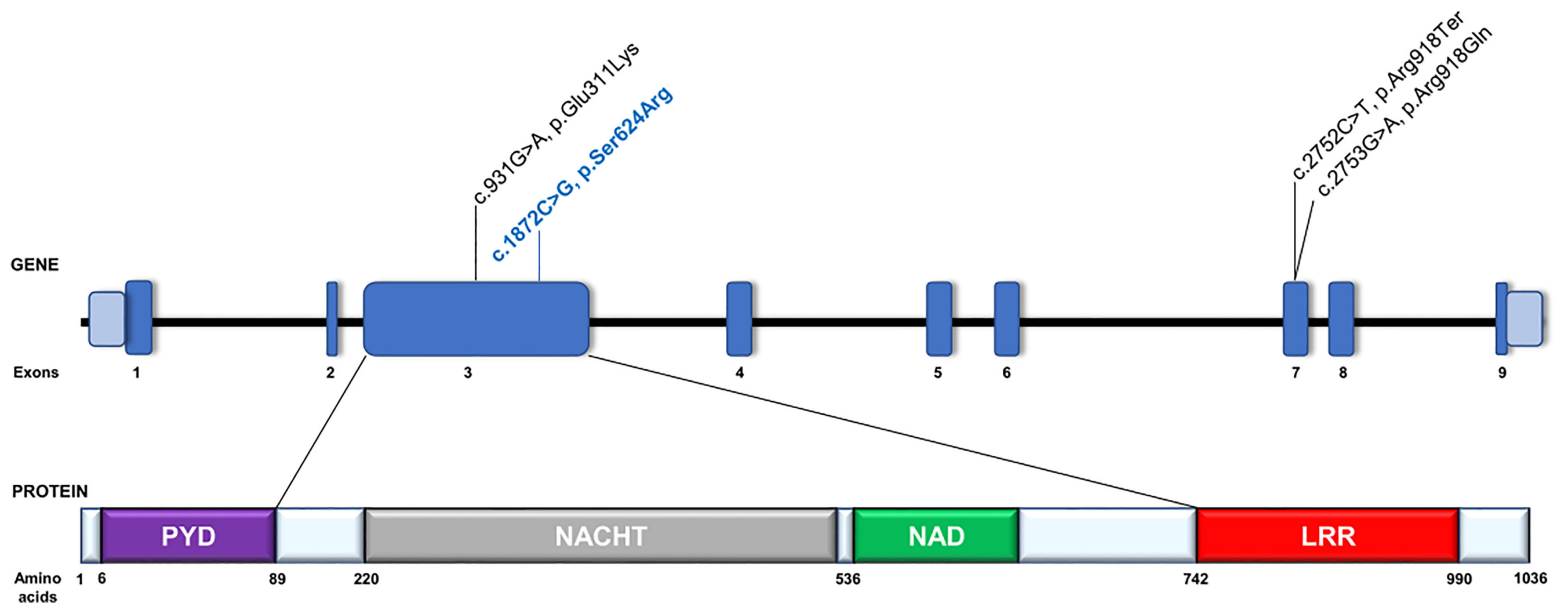
Schematic representation of the *NLRP3* gene and its protein organization. Gene and protein structures are depicted based on the canonical transcript NM_001243133.1 and reference protein sequence NP_001230062.1. The NRLP3 protein consists of an N-terminal pyrin domain (PYD), a central nucleotide-binding oligomerization domain (NACHT), a NACHT-associated (NAD) domain, followed by a leucine-rich repeat (LRR) domain at the C terminus. Previously reported *NLRP3* pathogenic variants involved in DFNA34 or atypical NLRP3-AID development are in black text. The c.931G>A, p.Glu311Lys variant was previously reported as c.937G>A, p.Glu313Lys based on NM_001127462.1 reference sequence (13). The variant identified in this study is labelled in blue text.

To search for the genetic cause of HL in the studied family, we analyzed 237 genes known for their involvement in isolated and syndromic forms of HL. After performing the multi-gene HL panel, we found only one variant located in the *NLRP3* gene that could explain the development of HL. The heterozygous transversion NM_001243133.1:c.1872C>G in exon 3 of the *NLRP3* gene (**Figure 1B**) has not been reported in population databases (**Table 1**). It is predicted to result in a missense substitution NP_001230062.1:p.Ser624Arg, which corresponds to the NACHT-associated domain (NAD) of the NLRP3 protein (**Figure 2**). The variant first detected in the proband, completely segregated with HL in the family, and was found in six other HL-affected individuals (**Figure 1A**). Based on the available data, the detected *NLRP3* change was classified as likely pathogenic (**Table 1**).

**Table 1:** Characteristics of the *NLRP3* variant detected in this study.

Variant cDNA Level	Variant Protein Level	Exon	Reference SNP ID	Population Frequencies			Pathogenicity Predictions						
				gnomAD	UK10K	EVS	SIFT	PolyPhen-2	Mutation Taster	LRT	CADD	REVEL	ACMG Classification*
c.1872C>G	p.Ser624Arg	3	N/A	0	0	0	T (0.053, 0.058, 0.056)	B (0.409)	P (0.6495)	N (0.002316)	D (24.6)	0.6129	LP (PM2, PP1_moderate, PP4_supporting, PS3_supporting )

*ACMG classification criteria legend: LP, likely pathogenic; PM2, moderate pathogenicity evidence; PP1_moderate, moderate pathogenicity evidence; PP4_supporting, supporting pathogenicity evidence; PS3_supporting, supporting pathogenicity evidence; N/A, no data available; B, benign; D, damaging; N, neutral; P, polymorphism; T, tolerated.

### Patients With the p.Ser624Arg *NLRP3* Mutation Present Increased NLRP3 Inflammasome Activity *ex vivo*

**Figure 3:**
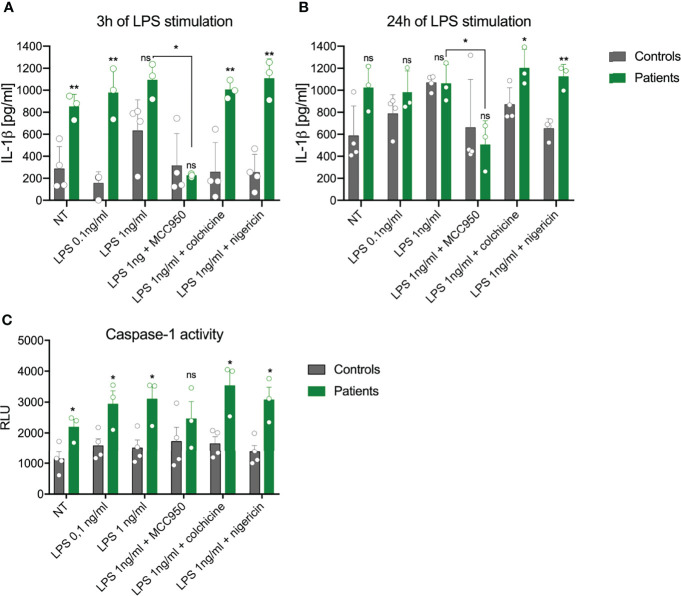
Impact of *NLRP3* mutation on inflammasome activation in peripheral blood mononuclear cells (PBMCs). The capacity of PBMCs from healthy controls (grey) and patients (green) to secrete IL-1β after stimulation with LPS or LPS+nigericin was analyzed after **(A)** 3 h or **(B)** 24 h The specific NLPR3 inhibitor MCC950, but not the microtubule inhibitor colchicine, was able to inhibit IL-1β release. **(C)** Activation of caspase-1 in PBMCs was evaluated after 24 h (mean ± SEM, results compared by *t*-test: *p < 0.05; **p < 0.01). NT, not treated; ns, non significant.

As the NLRP3 inflammasome is responsible for activation of caspase-1 and subsequent release of IL-1β, we compared the responses of PBMCs from patients and healthy controls to LPS stimulation *ex vivo*. Spontaneous release of IL-1β was almost 3-fold higher after 3 h of incubation, whereas this difference was less evident after 24 h of incubation (**Figures 3A, B**). Stimulation with 0.1 ng/ml of LPS for 3 h induced a significantly higher release of IL-1β from the cells with *NLRP3* mutation than from control cells (977.3 ± 231 pg/ml vs. 156.0 ± 103 pg/ml, **Figure 3A**). Extended stimulation time or higher LPS concentration decreased this difference. However, when cells treated with a higher concentration of LPS were stimulated with a second signal activator (nigericin), PBMCs from patients released significantly more IL-1β after both 3 h and 24 h of stimulation than the control cells (**Figures 3A, B**). Pretreatment of the PBMCs with the NLRP3 specific inhibitor MCC950, but not with colchicine, which indirectly suppresses NLRP3 inflammasome activity, significantly reduced the release of IL-1β in patient samples but not in controls (**Figures 3A, B**).

Additionally, we measured the total activity of caspase-1 in both cells and culture medium using an enzymatic activity test. Some 24 h after the start of the experiment, cells bearing the *NLRP3* mutation showed increased caspase-1 activity, which was also higher after LPS stimulation in comparison to control cells (**Figure 3C**). Upon addition of nigericin or colchicine, higher activity of caspase-1 remained. Pretreatment with MCC950 decreased (but not significantly) the response of patient-derived cells to LPS, while no effect was seen in control cells (**Figure 3C**).

### Clinical Reanalysis Reveals Phenotypic Features Related to an Autoinflammatory Disease Spectrum (NLRP3-AID/CAPS)

The proband from the family investigated in this study was first diagnosed with non-syndromic, progressive ADHL. Her HL was not associated with malformations of the external ear or other organs. She did not complain of medical problems involving organ systems other than the auditory system. After receiving the results of her genetic tests, which showed a probably causative variant in the *NLRP3* gene, we performed a clinical reanalysis of the affected individuals, focused now on the symptoms and signs of NLRP3-AID. ARTA showed progressive bilateral HL; the calculated ATD was significant at all frequencies and ranged from 0.82 dB/year (4 kHz) to 1.45 dB/year (2 kHz) (**Figure 1C**). All three subjects (II.6, III.7, III.8) admitted having episodes of conjunctivitis, which were more frequent in the proband’s mother and stepsister but occurred only occasionally in the proband. The stepsister also reported recurrent uveitis and a couple of stress-triggered episodes of unexplained fever. The proband’s mother complained of arthralgia and myalgia. All had elevated laboratory inflammatory markers, such as C-reactive protein (CRP) and serum amyloid A (SAA), and in some laboratory examinations mild blood leukocytosis, neutrophilia, and increased erythrocyte sedimentation rate were also noted (**Table 2**). After refining the clinical picture in the light of the Eurofever/Printo classification criteria, we diagnosed the patients as having NLRP3-AID (14), and therapy with IL-1 receptor antagonist (anakinra) was introduced.

**Table 2:** Inflammatory markers level.

Patients	Before anakinra			After 3 mo. of anakinra		
Inflammatory markers*	ESR	CRP	SAA	ESR	CRP	SAA
II.6	16	0.7	5.04	11	0.5	4.0
III.7	15	2.5	17.9	3	0.2	1.8
III.8	21	0.9	2.1	6	0.1	0.4

*Inflammatory markers done outside flares.

ESR mm/h ref.0-10, CRP mg/dl ref.0-0.8, SAA mg/dl ref. < 0.64.

**Figure 4:**
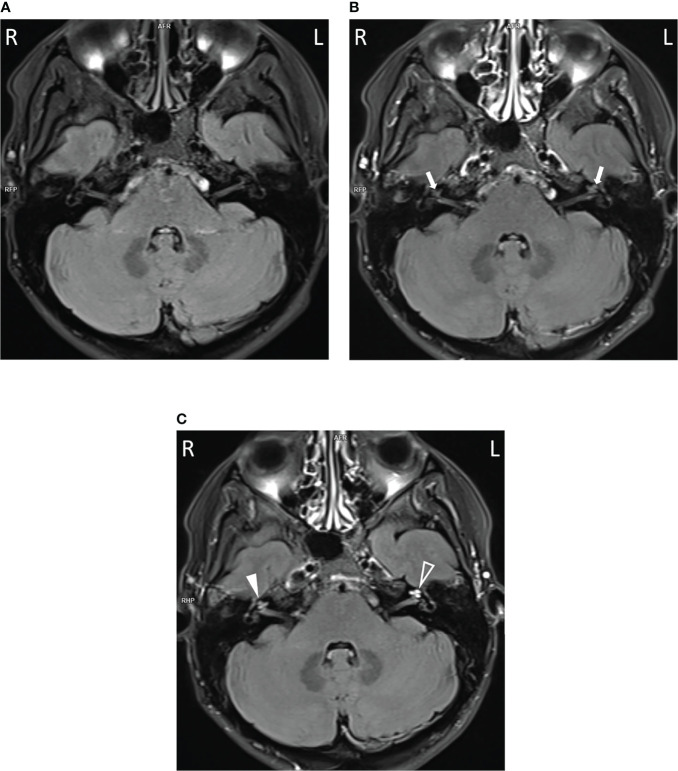
Cochlear enhancement on FLAIR images in the proband. In comparison to non-contrast image **(A)**, mild cochlear enhancement (arrows) is seen on the early FLAIR image **(B)**. On the late FLAIR image **(C)**, substantial enhancement (more pronounced on the left, empty arrowhead) is observed in both cochleas (arrowheads).

**Figure 5:**
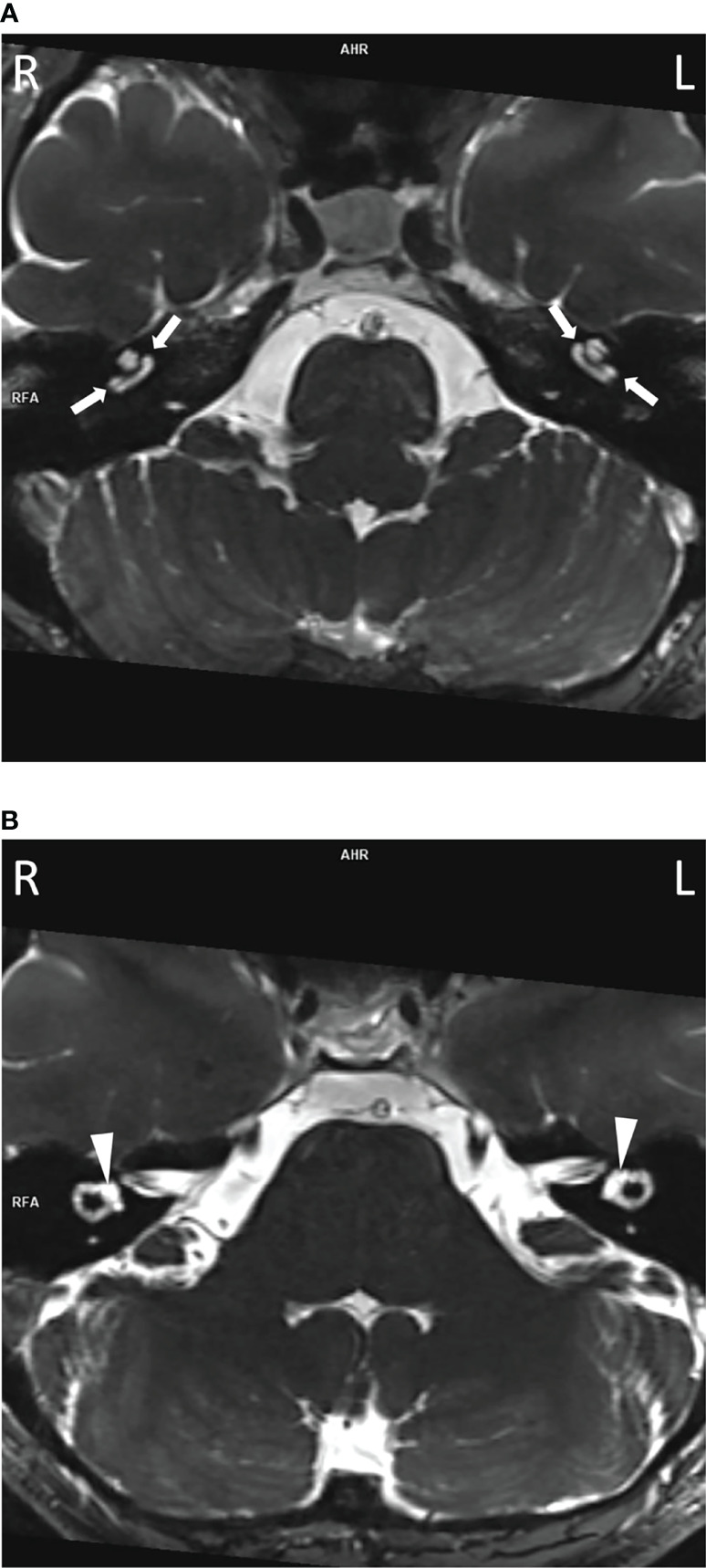
The decreased bright cochlear fluid signal on T2-weighted images in the proband. Slight diminishing of the bright fluid signal was visible in both cochleas (**A**, arrows) compared to vestibules (**B**, arrowheads) and more pronounced on the left.

To assess their auditory system, an MRI was performed. In all three subjects, pathologically strong cochlear enhancement on late FLAIR images (performed approx. 75 min after contrast administration) was observed in all turns of both cochleas (albeit asymmetrically). On the early FLAIR images (performed approx. 15 min after contrast administration), cochlear enhancement was less pronounced and was not uniformly distributed in all turns (**Figures 4A–C**). In the proband, a subtle decrease of the bright signal on T2-weighted images (CISS sequence) within both cochleas was found; moreover, a subtle reduction in the signal on T2-weighted images was more pronounced on the same side as where stronger enhancement was seen on FLAIR images (**Figures 5A, B**).

## Discussion

In this study, we have identified a novel *NLRP3* variant and, after combining genetic and functional data, we have demonstrated that it is causative of hereditary HL, with an autosomal dominant mode of inheritance. HL predominated in the investigated family’s clinical picture, but targeted phenotypic reanalysis also revealed minor inflammatory symptoms. The collected clinical data supported by serologic evidence of inflammation allowed us to diagnose that the patients suffered from an NLRP3-AID – although the presented features are insufficient to diagnose any particular NLRP3-AID condition such as Muckle–Wells syndrome (MWS), neonatal-onset multisystem inflammatory disease (NOMID), or familial cold autoinflammatory syndrome (FCAS) (5). After evaluating the available literature, we find that only four other families have been reported as having either isolated HL (DFNA34) or HL as a prominent feature of an atypical NLRP3-AID phenotype.

In one Chinese family with an *NLRP3* mutation (NM_001127462.1:c.937G>A, NP_001120934.1:p.Glu313Lys) previously linked to MWS, the proband and two other family members had isolated HL, but the other six HL individuals had minor MWS-related inflammatory symptoms such as conjunctivitis and uveitis, oral ulcers, arthralgia and arthritis, and erythematous rash, occurring in different combinations (13). An atypical form of NLRP3-AID was also recognized in one of two families presented by Nakanishi et al. (2), which carried the same *NLRP3* missense mutation (NM_001243133.1:c.2753G>A, NP_001230062.1:p.Arg918Gln). In one of the two families, a syndromic form of HL was reported, but the autoinflammatory phenotype (including episodic urticaria, periodic fevers, conjunctivitis, oral ulcers, cervical lymphadenopathy, arthritis, arthralgia, bursitis, headaches) did not meet the diagnostic criteria for MWS, NOMID, or FCAS. In the second family (North American Caucasian), HL occurred together with either multiple sclerosis or some nonspecific signs and symptoms, and it was regarded as isolated HL (DFNA34) (2). In 2021, Kim et al. described two affected family members who had a second DFNA34 pedigree due to an *NLRP3* mutation (NM_001243133.1:c.2752C>T, NP_001230062.1:p.Arg918Ter) (15) (**Figure 2**).

HL in NLRP3-AID is considered a common clinical manifestation. It is typically observed in NOMID and MWS but only rarely in FCAS (16). In the defined NLRP3-AID syndromes and in atypical phenotypes or in DFNA34, some general features of HL remain similar: in these patients, HL is usually sensorineural, more often affects the higher frequencies, and gradually progresses over time (16, 17). This was also the case for the family reported in this study, although the age at HL onset, the severity of hearing deterioration, and its rate of progression vary among NLRP3-AID patients. The onset of HL may range from the first months to the fourth decade of life, and individuals can have severe to profound HL (15, 17). In the family described here, the onset of HL was after 15 years of age and HL had progressed over time. In none of the subjects it had reached the point where cochlear implants were needed.

Based on the current understanding of the pathogenesis of HL in NLRP3-AID, the condition arises as a consequence of local cochlear autoinflammation. Recent studies have demonstrated that immune cells (macrophage/monocyte-like cells) in the cochlea can activate their NLRP3 inflammasome. If this activation is aided by an existing *NLRP3* mutation (an underlying cause of NLRP3-AID), abnormal cochlear activation of the NLRP3 inflammasome may occur. The result may be cochlear inflammation accompanied by progressive hearing deterioration (2, 18). A strong argument in favor of cochlear inflammation being causally involved in the development of HL in NLRP3-AID, is the pathological enhancement visible on the MRI post-contrast FLAIR images of the cochlea. Enhancement indicates that the contrast material has diffused into cochlear tissues from the blood vessels, made more permeable by inflammation (15). In previous studies and in our patients, pathological cochlear enhancement has been associated with the presence of HL (16, 19). Here, we found that the late post-contrast FLAIR images were more sensitive than the early ones in detecting cochlear inflammation. The differences observed in the degree of cochlear enhancement may correspond to differences in the magnitude or stage of the local inflammation.

Overproduction of IL-1β, considered the central mediator of inflammation, plays a main role in the pathogenesis of NLRP3-AID; consequently, treatment with anti-IL-1 receptor inhibitors such as anakinra or canakinumab is recommended for this group of patients (5). A positive therapeutic effect on hearing (such as its stabilization in the majority of patients or improvement in some individuals) has also been achieved, especially when treatment is begun early (15, 19–23). Poor response to anti-IL-1 receptor inhibitors is likely explained by a chronic inflammation that has already caused irreversible cochlear damage. Of the four families with atypical NLRP3-AID or DFNA34, this form of therapy has only been introduced in one family with syndromic HL. Over a 5-month follow-up period, it restored normal hearing thresholds in the children and improved the hearing of one adult (2). In three of our patients, subcutaneous anakinra at a dose of 100 mg daily was administered; it appeared to be well-tolerated and we expect to be able to assess the efficacy of this therapy within the next couple of months.

In our work, we also wanted to find out how the novel *NLRP3* variant affects the function of the NLRP3 inflammasome, a critical component of the inflammatory signaling pathway in the innate immune system. For this purpose, we tested the circulating monocytes from the patients for the effects of the identified *NLRP3* mutation on IL-1β release. In contrast to a previous report, unstimulated PBMCs from our patients secreted more IL-1β after 3 h of incubation than did control cells (24). The most discriminatory condition between mutant and control PBMCs was found to be 3 h of stimulation with a low dose of LPS. In most human cells, activation of the NLRP3 inflammasome requires two signals: priming *via* the TLR receptor, which upregulates the expression of inflammasome components, and a second signal (e.g. potassium efflux or lysosomal leakage) which induces assembly of the NLRP3 inflammasome. However, in human monocytes LPS binds the TLR4 receptor, which triggers activation of the NLPR3 inflammasome through an alternative pathway. This mechanism explains why only one-signal stimulation reveals differences in the NLRP3 hyperactivation mutation (25). A similar condition of 3 h of LPS stimulation was reported by Rieber et al. (24) to be most accurate in testing *NLRP3* mutations. It is probable that monocytes from NLRP3-AID patients activate NLPR3 more rapidly (24, 26). The two-signal stimulation (with LPS and nigericin) did not further increase the concentration of IL-1β, which is in line with other studies on NLRP3-AID (24, 27). Such conditions decrease the amount of IL-1β released by monocytes from healthy controls, probably due to rapid induction of pyroptosis while having less effect on the patients’ cells but this assumption requires further studies. Longer stimulation times decrease the observed differences in IL-1β due to increased secretion by control cells. Pre-treatment of cells with the NLRP3 specific inhibitor confirmed the role of NLRP3 in the secretion of IL-1β. Intriguingly, the capsase-1 activity test did not reflect the observed changes in the IL-1β release, limiting its usefulness in such experimental settings.

Colchicine, which has been shown to inhibit NLRP3 inflammasome formation in macrophages (28), was ineffective in blocking the IL-1β release after LPS stimulation. We also measured the activity of caspase-1, which increased even in unstimulated PBMCs from NLRP3-AID patients. This assay was performed only after 24 h of stimulation, and in each tested condition, PBMCs from NLRP3-AID patients showed higher activity of caspase-1 in comparison to control samples (except for the MCC950-treated cells). However, the lack of evident inhibition of caspase-1 activity by the MCC950 inhibitor suggests that further studies applying other techniques of caspase analysis in these patients should be performed. Nevertheless, these results support the involvement of NLRP3 in this process and show that the analyzed c.1872C>G (p.Ser624Arg) genetic variant in our patients resulted in increased activity of the NLRP3 inflammasome. NLRP3-AID is a consequence of a heterozygous hyperactivating *NLRP3* mutation which leads to excessive NLRP3 inflammasome activation. Similarly, as in our patients, most of the variants causative of NLRP3-AID are missense variants which accumulate in the part of the gene that encodes the NACHT and NAD domains of the NLRP3 protein (5). Mutation in this same gene region was detected in the Chinese family, which showed a variable phenotypic expression of DFNA34/MWS (13). In contrast, the two gene variants detected in the DFNA34 patients locate more toward the 3′ end (**Figure 2**) which encodes the LRR domain. Based on previous findings, it has been proposed that the *NLRP3* pathogenic variants affecting the LRR domain might be related to a milder phenotype (2, 15); this proposal might be expanded to indicate that the milder phenotype is also observed for *NLRP3* mutations affecting other parts of the gene.

In summary, the results of our study highlight the need to perform genetic testing in patients with HL. They show that the multi-organ autoinflammatory phenotype of NLRP3-AID may in fact be considered an “isolated” HL accompanied by marginal and difficult to recognize symptoms that can easily be overlooked. Efforts to identify patients carrying an *NLRP3* mutation have tangible practical value. They may help to explain not only the cause of HL but also other medical problems that have been initially neglected, and, uncommonly for genetically determined HL, the patients can be offered an effective pharmacological therapy. Individuals with NLRP3-AID should also be made aware of the variable expressivity of the disorder.

## Data Availability Statement

The raw data supporting the conclusions of this article will be made available by the authors, without undue reservation.

## Ethics Statement

The studies involving human participants were reviewed and approved by Ethics committee at the Institute of Physiology and Pathology of Hearing. The patients/participants provided their written informed consent to participate in this study.

## Author Contributions

DO and MLL performed genotyping and computational analysis. MO, DO, EW-S, MF, MOr, NB and HS participated in phenotyping and clinical data collection. AJ and TS performed functional studies. MO, DO, and TS analyzed the data and wrote the manuscript. All authors read and approved the final manuscript.

## Funding

This work was funded by a Sonata BIS6 grant of the National Science Centre 2016/22/E/NZ5/00470 (MO). TS and AJ were funded by the Polish National Science grant UMO-2016/23/D/NZ6/02554.

## Conflict of Interest

The authors declare that the research was conducted in the absence of any commercial or financial relationships that could be construed as a potential conflict of interest.

## Publisher’s Note

All claims expressed in this article are solely those of the authors and do not necessarily represent those of their affiliated organizations, or those of the publisher, the editors and the reviewers. Any product that may be evaluated in this article, or claim that may be made by its manufacturer, is not guaranteed or endorsed by the publisher.
